# Supplementary material for: Why Is the Correct Selection of Trichoderma Strains Important? The Case of Wheat Endophytic Strains of T. harzianum and T. simmonsii
Source: J Fungi (Basel). 2021 Dec 17;7(12):1087. doi: 10.3390/jof7121087 (PMC8704890; doi:10.3390/jof7121087)
Supplement: Supplementary file 1 [file jof-07-01087-s001.zip › jof-1453514-supplementary.pdf]

Table S1. Growth of 54 fungal isolates, obtained from the root endosphere of wheat Berdun, in potato dextrose broth (PDB) medium with 0, 10, 20, 30, or 40% (vol/vol) polyethylene glycol (PEG). The genus was assigned by analyzing of the ITS1-ITS4 region sequence.

Isolate	Genus	PDB	PDB+10% PEG	PDB+20% PEG	PDB+30% PEG	PDB+40% PEG
1	*Fusarium*	++	+++	++	++	++
2	*Fusarium*	+++	+++	++	++	++
3	*Rhizopus*	+++	+++	+++	+++	+++
4	*Microdochium*	+++	++++	++++	++	+
5	*Fusarium*	-	-	-	-	-
6	*Alternaria*	+++	+++	++	+	+
7	*Aspergillus*	++	++	+++	+++	+++
8	*Penicillium*	++	+++	++++	++++	+++
9	*Fusarium*	+	+	+	+	+
10	*Monosporascus*	+++	++	+	-	-
11	*Alternaria*	+	+	+	+	+
12	*Actinomucor*	+++	+++	++	++	++
13	*Fusarium*	-	-	-	-	-
14	*Monosporascus*	-	-	-	-	-
15	*Actinomucor*	++	++	++	+	+
16	*Monosporascus*	-	-	-	-	-
17	*Sordaria*	+	+	++	+	-
18	*Macrophomina*	+++	++	++	+	+
19	*Alternaria*	++	++	++	+	-
20	*Alternaria*	+	+	+	+	+
21	*Fusarium*	++	++	++	++	++
22	*Alternaria*	+	+	+	+	-
23	*Periconia*	-	-	-	-	-
24	*Alternaria*	++	++	+	+	-
25	*Fusarium*	+	+	+	+	+
26	*Fusarium*	+	+	+	+	+
27	*Fusarium*	+	+	+	+	+
28	*Fusarium*	-	-	-	-	-
29	*Trichoderma*	+++	++	++	+	+
30	*Fusarium*	++	++	++	+	+
31	*Fusarium*	++	+	+	+	-
32	*Trichoderma*	+++	++	+	+	+
33	*Waitea*	+	+	-	-	-
34	*Fusarium*	-	-	-	-	-
35	*Fusarium*	+	-	-	-	-
36	*Aspergillus*	+++	+++	++	+	-
37	*Diaporthe*	+	+	-	-	-
38	*Setophoma*	-	-	-	-	-
39	*Chaetomium*	-	-	-	-	-
40	*Monosporascus*	-	-	-	-	-
41	*Periconia*	-	-	-	-	-
42	*Monosporascus*	-	-	-	-	-

43	*Monosporascus*	-	-	-	-	-
44	*Fusarium*	+	+	+	-	-
45	*Monosporascus*	-	-	-	-	-
46	*Fusarium*	-	-	-	-	-
47	*Fusarium*	-	-	-	-	-
48	*Fusarium*	-	-	-	-	-
49	*Trichoderma*	++	++	+	+	+
50	*Fusarium*	+	+	+	-	-
51	*Alternaria*	+	+	-	-	-
52	*Fusarium*	-	-	-	-	-
53	*Sordaria*	++	++	+	+	-
54	*Trichoderma*	+++	+++	+++	++	+

Data scala: - (no growth), + to +++ (increasing hyphal growth), and ++++ (completely bushy well).

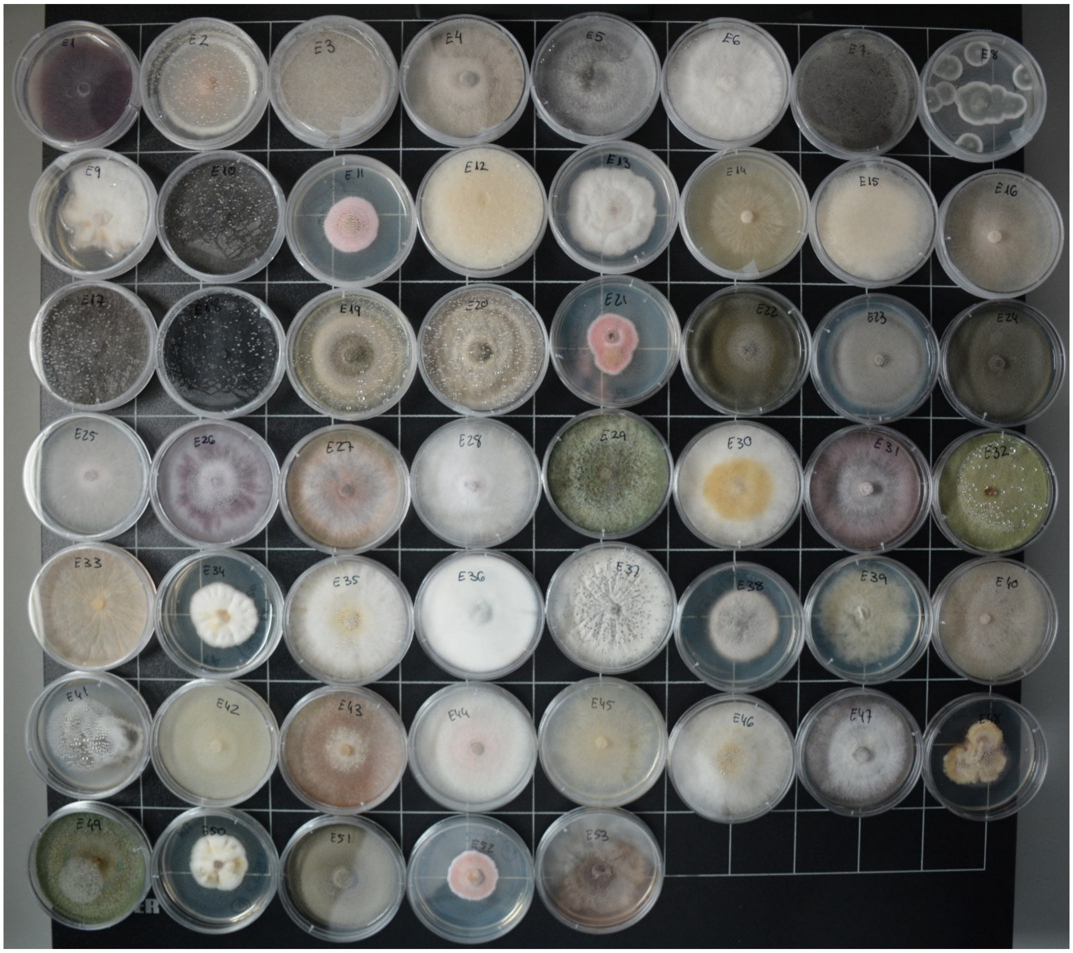

Figure S1. Potato dextrose agar (PDA) cultures of the 53 fungal endophytes that were obtained in this study.
